# ATP-Sensitive Potassium Channels Mediate the Cardioprotective Effect of *Panax notoginseng Saponins* against Myocardial Ischaemia–Reperfusion Injury and Inflammatory Reaction

**DOI:** 10.1155/2020/3039184

**Published:** 2020-10-20

**Authors:** Ke Ning, Li Jiang, Ting Hu, Xingyu Wang, Aihua Liu, Yimin Bao

**Affiliations:** ^1^Department of Physiology, School of Basic Medical Sciences, Shanghai University of Traditional Chinese Medicine, No. 1200 Cailun Road, Shanghai, China; ^2^Experimental Center, School of Basic Medical Sciences, Shanghai University of Traditional Chinese Medicine, No. 1200 Cailun Road, Shanghai, China

## Abstract

Inflammatory response during myocardial ischemia reperfusion injury (MIRI) is essential for cardiac healing, while excessive inflammation extends the infarction and promotes adverse cardiac remodeling. Understanding the mechanism of these uncontrolled inflammatory processes has a significant impact during the MIRI therapy. Here, we found a critical role of ATP-sensitive potassium channels (K_ATP_) in the inflammatory response of MIRI and its potential mechanism and explored the effects of Panax Notoginseng Saponins (PNS) during this possess. Rats underwent 40 min ischemia by occlusion of the left anterior descending (LAD) coronary artery and 60 min of reperfusion. PNS was treated at the corresponding time point before operation; 5-hydroxydecanoate (5-HD) and glybenclamide (Gly) (or Nicorandil (Nic)) were used as pharmacological blocker (or nonselective opener) of K_ATP_. Cardiac function and pathomorphology were evaluated and a set of molecular signaling experiments was tested. K_ATP_ current density was measured by patch-clamp. Results revealed that in MIRI, PNS pretreatment restored cardiac function, reduced infarct size, and ameliorated inflammation through K_ATP_. However, inhibiting K_ATP_ by 5-HD and Gly significantly reversed the effects, including NLRP3 inflammasome and inflammatory mediators IL-6, MPO, TNF-*α*, and MCP-1. Moreover, PNS inhibited the phosphorylation and nuclear translocation of NF-*κ*B in I/R myocardium when the K_ATP_ was activated. Importantly, PNS promoted the expression of subunits and activation of K_ATP_. The study uncovered K_ATP_ served as a new potential mechanism during PNS modulating MIRI-induced inflammation and promoting injured heart recovery. The manipulation of K_ATP_ could be a potential therapeutic approach for MIRI and other inflammatory diseases.

## 1. Introduction

Coronary heart disease (CHD) is a major disease with high morbidity and mortality worldwide [1]. After acute myocardial infarction, the use of thrombolytic therapy or primary percutaneous coronary intervention is the most effective strategy for reducing myocardial infarct and improving clinical outcomes. However, during this period, it will inevitably cause myocardial ischemia-reperfusion injury (MIRI). Although MIRI is not always immediately lethal, it often leads to delayed cardiomyocyte death by necrosis and uncontrolled inflammation, causing permanent injury to the myocardium.

Studies have shown that ischaemic preconditioning (IPC) [2] and pharmacological preconditioning can significantly alleviate MIRI [3] and that the opening of the K_ATP_ channel plays an important role in their endogenous cardioprotection mechanisms [4]. However, it is not clear that the role of K_ATP_ protects myocardium from inflammation induced by MIRI.

K_ATP_ channels are heterooctameric membrane protein complexes composed of four inward-rectifier potassium channel 6 (Kir6, either Kir6.1 or Kir6.2) subunits and four ABCC (ATP-binding cassette, subfamily C) family member sulfonylurea receptor (SUR, as SUR1, SUR2A, or SUR2B) subunits, with a total molecular weight of approximately 880 kDa [5]. K_ATP_ channels can be classified into the sarcolemmal (sarcK_ATP_) and mitochondrial (mitoK_ATP_) types. Studies have suggested that K_ATP_ channels are involved in the inhibition of ischemia–reperfusion (I/R) insult, which has a dependent mechanism, and demonstrated that the activation of these channels suppresses calcium overload and cell swelling during I/R [6]. However, whether sarcK_ATP_ or mitoK_ATP_ plays a leading role in such cardioprotection is still under debate.

The inflammatory cascade is an important therapeutic target for the treatment of both acute ischemia injury and repair of the myocardium [3, 7]. It is associated with a significant production of an array of mediators, including chemokines, proteases, TNF-*α* [8], and cytokines, such as interleukins (ILs) [9]. The inflammatory reaction has been noted in MIRI and used as a parameter for assessing cardiac damage after MIRI [10]; this means that greater excessive inflammatory response results in more severe myocardial injury after I/R. Thus, investigating the effect and alterations of inflammatory factors involved in I/R injury is crucial to our understanding of this process and resulting clinical therapies. Reports have demonstrated that K_ATP_ channels play significant roles in inflammation-mediated cerebral I/R injury, leading K_ATP_ channels to be regarded as a necessary target [11], and I/R-induced arrhythmias in STZ-induced diabetic rats [12]. However, the mechanism of how K_ATP_ channels mediate cardioprotection against the inflammatory reaction to MIRI is still unknown. These channels' overall effects on I/R-induced inflammation and the underlying mechanism involved in cardioprotection require further investigation.

Phytopharmaceuticals are becoming essential in modern and traditional medicine owing to their nontoxic nature. PNS is a major active ingredient of the traditional Chinese herb *Panax notoginseng*. Clinical research has demonstrated that steroidal saponins may act as the antioxidation, the reduction of intracellular Ca^2+^ overloading, and anti-inflammatory activity [13, 14]. Nevertheless, the mechanism of the protective effect of PNS on I/R in the heart is still unclear, especially with respect to the role of K_ATP_ channels.

Therefore, we investigated how K_ATP_ channels mediate the cardioprotection of PNS against I/R-induced inflammatory reaction using a rat MIRI model and explored its underlying mechanisms.

## 2. Materials and Methods

### 2.1. Animals and Drugs

Male adult Sprague–Dawley rats (8 weeks, mean body weight mass 250 ± 20 g) were purchased from Shanghai Slack Laboratory Animal Company (Certificate No. SCXK (Shanghai) 2012-0002). All procedures in this study were approved by the Animal Care and Use Committee of Shanghai University of Traditional Chinese Medicine and were performed in accordance with the guide for the care and use of laboratory animals published by the National Institutes of Health (NIH Publications No. 8023, revised 1978). Rats were housed in cages at a temperature of 23°C ± 2°C and relative humidity of 55% ± 10% under a 12 h/12 h light–dark cycle; they were given ad libitum access to water and a standard laboratory diet. PNS was purchased from Kunming Pharmaceutical Group Co., Ltd. (Yunnan, China) under the clinical name of Xueshuantong Injection. Nicorandil (N35390, K_ATP_ channel opener), glybenclamide (G0639, nonselective K_ATP_ channel blocker), and 5-hydroxydecanoic (H135, selective mitoK_ATP_ channel blocker) were purchased from Sigma Aldrich (MO, USA).

### 2.2. Myocardial I/R Model and Medicine Treatment

**Figure 1:**
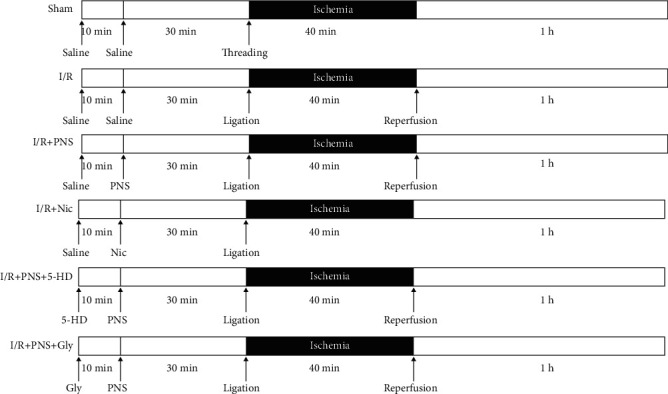
The operation process of animals and the time of each drug. Schematic illustration of the experimental process regarding I/R model protocol and drug treatment.

The animals were randomly divided into six groups: sham, I/R, I/R with PNS (I/R+PNS), I/R with nicorandil (I/R+Nic), I/R with PNS and 5-hydroxydecanoate (I/R+PNS+5-HD), and I/R with PNS and glybenclamide (I/R+PNS+Gly) (*n* = 17–20 in sham, I/R and I/R+PNS group; *n* = 12–15 in other groups). Rats were anesthetized and placed in a supine position. Thoracotomy was performed through an incision of the left second and third ribs to expose the heart, and 5-0 suture silk was passed through the myocardium around the left anterior descending coronary artery, which was 1–2 mm under the boundary of the pulmonary conus and left auricle. The suture silk was ligated for 40 min to induce myocardial ischemia and then released, allowing reperfusion for 60 min. Sham-operated animals underwent the same procedure as the I/R group did without ligation of the silk. The animals were intraperitoneally injected with saline as well as PNS (50 mg/kg) [15] or nicorandil (Nic; 0.3 mg/kg) [16] 30 min before ischemia, and 5-HD (10 mg/kg) [17] or Gly (5 mg/kg) [18] was intraperitoneally administered 40 min before ischemia (see Figure 1).

### 2.3. Hemodynamic Measurements

Prior to surgical modelling, a catheter filled with heparinized (10 U/mL) saline was inserted into the left ventricle along the right carotid artery, and the other end was connected to a bioinformatics processing system (RM6240BD, Chengdu, China) by using a pressure transducer. The left ventricular pressure curve was continuously recorded during the experiment, and the hemodynamic parameters, including the mean left ventricular systolic pressure (mLVSP), mean left ventricular diastolic pressure (mLVDP), maximum rise rate of left ventricular pressure (+dp/dt_max_), and maximum fall rate of left ventricular pressure (−dp/dt_max_), were measured at baseline, preischemia, 40 min postischemia, and 60 min postreperfusion.

### 2.4. Histological Procedure

At the end of reperfusion, the myocardium below the ligature (0.7 cm × 0.3 cm × 0.3 cm) was removed from the left ventricle of rats and prefixed by 4% paraformaldehyde at 4°C. The specimens were then fixed and embedded in paraffin after dehydration in different concentrations of ethanol before being sectioned into ultrathin slices for staining with hematoxylin and eosin (H&E). The structure of the myocardium was observed with an optical microscope and photographed.

### 2.5. Myocardial Infarct Size Determination

At the end of the I/R or drugs treatment protocol, LAD was occluded again at the same occlusion site as the one during I/R. 1% Evan's blue dye was perfused via the right common carotid artery, and the heart was removed rapidly after the animal's lips were blue stained. The heart was frozen at -20°C 15 min and then cutted into 1~2 mm slices. Then, 2,3,5-triphenyltetrazolium chloride (TTC) was added to each slice for 10 min at 37°C. The area with viable tissues was seen in red, whereas the infarct area was seen in white. The infarct size was determined from the area that was not stained with Evan's blue and TTC. The infarct size was measured using the image tool software version 3.0 and was calculated using formula as reported previously [19].

### 2.6. Colorimetry and ELISA

The apical portion of myocardial tissue was taken, homogenized, and then used to form a 5% suspension. The detection procedure was conducted according to the manufacturer's instructions for the myeloperoxidase (MPO) assay kit used (Nanjing Jiancheng, A044). The optical density (OD) value was measured using a spectrophotometer. The enzyme activity unit was defined as the decomposition of 1 *μ*mol of H_2_O_2_ into one enzyme activity unit per gram of the sample in a 37°C water bath reaction system. Protein samples of myocardial tissue below the ligature were extracted using protein extraction kits (Thermo Fisher Scientific, USA). In brief, the mixture was homogenized and centrifuged at 3000 rpm at 4°C for 10 min. The resultant supernatant was taken as a whole protein. The contents of TNF-*α* and MCP-1 of the myocardium were assessed using ELISA (TNF-*α* Rat ELISA Kit, Life, KRC3011; MCP-1 Rat ELISA Kit, Life, KRC1011) and detected using a microplate reader according to the manufacturer's instructions.

### 2.7. Western Blotting and Real-Time Polymerase Chain Reaction Analysis

Whole protein was extracted. The concentration of whole protein was determined with a BCA protein assay kit (Beyotime, Shanghai, China) according to manufacturer instructions. The primary antibodies used were kir6.1 (Abcam, ab241996, 1 : 1000), kir6.2 (Santa Cruz, sc-390104, 1 : 1000), SUR1 (Abcam, ab32844, 1 : 200), SUR2 (Abcam, sc-25684, 1 : 500), NLRP3 (Proteintech, 19771-1-AP, 1 : 1000), IL-6 (Proteintech, 21865-1-AP, 1 : 1000), NF-*κ*B p65 (CST, 8242, 1 : 1000), and phospho-NF-*κ*B p65 (CST, 3033, 1 : 1000). Horseradish peroxidase–linked secondary antibodies were used to visualize the bound primary antibodies with chemiluminescence substrate ECL (Thermo, 34095). Protein was quantified through scanning densitometry in the X-film using Image-Pro plus 6.0. The densities of bands were quantified using an Image J Analysis System and expressed as ratios to GAPDH.

Total RNA was extracted from myocardial tissues with Trizol® reagent (Invitrogen, CA, USA) and reverse-transcribed with a SuperScript reverse transcriptase kit (Takara, Otsu, Japan). Gene expression was analyzed through a quantitative real-time polymerase chain reaction (PCR) using SYBR^∗^ dye (LightCycler^∗^96 Real-time PCR System). The target gene transcript level was determined relative to the signal from GAPDH and normalized to the mean value of samples from the sham group. The primers encoding rat (TNF-*α*), MCP-1, Kir6.1, Kir6.2, SUR1, SUR2, and GAPDH are presented in Table 1.

### 2.8. Immunofluorescence

Immunofluorescence (IF) was performed on tissue paraffin sections to determine the protein expression level of phospho-NF-*κ*B in the cardiomyocyte nuclei. Paraffin tissue sections were placed in xylene and gradient ethanol solutions (100%, 95%, 85%, and 75%) to deparaffinize and rehydrate the samples. Subsequently, antigen retrieval was performed by heating sections in citrate buffer (10 mM; pH 6.0) at 96°C–98°C for 10 min in a water bath. Furthermore, sections were pretreated with 0.5% TritonX-100, to increase cell membrane permeability and blocked with 10% normal goat serum for 30 min at room temperature to block any nonspecific binding. Sections were then reacted with primary antibodies against phospho-NF-*κ*B (CST, 3033, 1 : 100) at 4°C overnight and then incubated with secondary antibodies with fluorescence (Abcam Alexa Fluor 647, 1 : 400) for 2 h in the dark at RT. Slides were then counterstained with 4, 6-diamidino-2-phenylindole for 10 min at RT to reveal the nucleus. The stained sections were examined under a Leica fluorescence microscope.

### 2.9. Whole-Cell Patch-Clamp Recordings

After anaesthesia, the rats' hearts were removed rapidly, then mounted on the Langendorff perfusion apparatus, and then perfused using an aortic cannula. The perfusion solution was saturated with a mixture gas of 95% O_2_ and 5% CO_2_ at a flow rate of 5 mL/min at 37°C. First, the heart was perfused with calcium-free cell separation buffer for 10 min, which was then exchanged with low-calcium cell separation buffer containing 25 mg of type II collagenase (Worthington, LS004106), 5 mg of protease (Sigma, P8340), 50 *μ*M CaCl_2_, and 0.1% bovine serum albumin for 15 min. The ventricle was then cut into pieces in the low-calcium cell separation buffer to obtain ventricular myocytes. The successfully isolated myocytes were rod-shaped, with clear edges, contraction bands, and a smooth surface, which could be used for patch-clamp experiments. The cells were randomly divided into a control, I/R, I/R+low-dose PNS (50 mg/L), and I/R+high-dose PNS (100 mg/L) groups. The cell suspension was placed on the workbench of an inverted microscope (Olympus 70X). The Tyrode solution was perfused at a constant flow (1.5 mL/min). The extracellular medium consisted of 137 mM NaCl, 5.4 mM KCl, 1 mM MgCl_2_, 10 mM 4-(2-hydroxyethyl)-1-piperazineethanesulfonic acid, 10 mM glucose, 20 mM TEACl, and 0.001 mM nifedipine at pH 7.4 with NaOH. The intracellular medium consisted of 120 mM potassium L-aspartate (C_4_H_5_K_2_NO_4_), 20 mM KCl, 0.5 mM MgCl_2_, 10 mM HEPES, 10 mM egtazic acid, and 10 mM K_2_ATP at pH 7.2. Subsequently, cardiomyocytes underwent 10 min of KOH perfusion in the extracellular solution, followed by 3 min of perfusion in the ischaemic solution (118.0 mM NaCl, 4.7 mM KCl, 1.25 mM CaCl_2_, 1.2 mM KH_2_PO_4_, 1.2 mM MgSO_4_, 25.0 mM NaHCO_3_, 10 mM 2-deoxy-D-glucose, and 10 mM Na_2_S_2_O_4_) and 3 min of perfusion in the extracellular solution to record the K_ATP_ current. Changes in the K_ATP_ current were recorded using a whole-cell patch clamp. K_ATP_ currents were recorded and analyzed with pClamp 10.0 and Clampfit 10.0 software (Axon Instruments, Union City, CA, USA). Whole-cell current recordings of membrane currents were recorded using the patch-clamp technique of whole-cell configuration using a patch-clamp amplifier (Axopatch ID, Axon Instrument, Foster City, CA, USA). When the ramp voltage-clamp method was employed, an intelligent arbitrary function synthesizer (model 1731, NF Instruments, Yokohama, Japan) was used to supply the command pulse. Under voltage-clamp conditions, the clamp voltage was −40 mV, which was then depolarized from −100 mV to +80 mV at a rate of 20 mV/s, and the stimulation interval was 9 s. Data analysis was performed on the current density at a clamping voltage of 0 mV.

### 2.10. Statistical Analysis

The results were expressed at the mean ± SE. GraphPad prism 6 was used to analyze the data. Before data analysis, all variables were tested for normality and equal variance. The differences among multiple groups were analyzed by One-way ANOVA. *p* < 0.05 was considered statistically significant.

## 3. Results

### 3.1. PNS Attenuated MIRI and Recovered Cardiac Function

**Figure 2:**
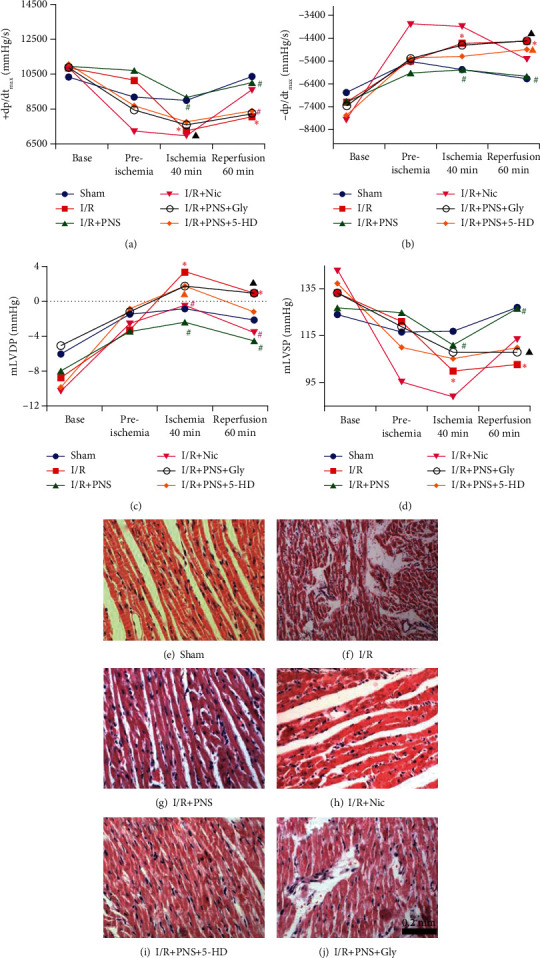
PNS pretreatment could improve hemodynamics and myocardial morphology in rats with MIRI. (a) Maximum rise rate of left ventricular pressure (+dp/dt_max_). (b) Maximum fall rate of left ventricular pressure (−dp/dt_max_). (c) Mean left ventricular diastolic pressure (mLVDP). (d) Mean left ventricular systolic pressure (mLVSP) (*n* = 12 − 15). (e–j) Myocardial histology according to H&E staining in different groups (*n* = 3). ^∗^*p* < 0.05 vs. Sham, #*p* < 0.05 vs. I/R, ^▲^*p* < 0.05 vs. I/R+PNS.

**Figure 3:**
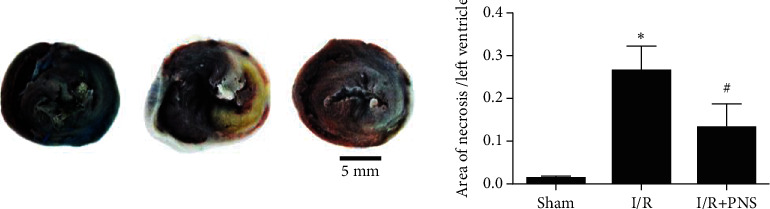
PNS reduced myocardial infarct size. The effect of with or without PNS (Sham, I/R, and I/R+PNS) on myocardial infarct size (*n* = 5). ^∗^*p* < 0.05 vs. Sham, #*p* < 0.05 vs. I/R; Scale bar = 5 mm.

To evaluate the effect of PNS on MIRI-induced cardiac functional alteration in vivo, a rat model of MIRI was introduced, hemodynamic measurements were taken, and a pathological section of the myocardium was assessed. As presented in Figures 2(a)–2(d), ischemia for 40 min caused a significant decline in +dp/dt_max_ and mLVSP as well as an increase in −dp/dt_max_ and mLVDP, indicating an impairment of cardiac systolic and diastolic function. PNS or Nic (nonselective K_ATP_ opener) exhibited better recovery of cardiac function after 60 min of reperfusion. They exhibited a beneficial effect on MIRI-induced left ventricular dysfunction, which was confirmed by the increased +dp/dt_max_ and decreased −dp/dt_max_ and LVDP (*p* < 0.05 vs. I/R group). By contrast, the administration of 5-HD (selective mitoK_ATP_ blocker) or Gly (nonselective K_ATP_ blocker) before PNS reduced cardiac function (*p* < 0.05 vs. I/R+PNS group). This was further supported by the H&E staining of the myocardium (Figures 2(e)–2(j)). Myocardial interstitial oedema, rupture of myocardial fibres, and infiltration of leukocytes were observed after MIRI. Nevertheless, treatment with PNS or Nic significantly reduced leukocyte infiltration and the swelling of myocardial fibres, but more severe myocardial damage was observed after 5-HD or Gly administration, suggesting that PNS could improve heart function and MIRI-induced cardiac pathological changes, which could be blocked by 5-HD and Gly. In addition, our results also showed that the PNS significantly reduced the myocardial infarct size, compared with the I/R group (Figure 3).

### 3.2. PNS Attenuated MIRI by Increasing Opening and Expression of K_ATP_ Channels

**Figure 4:**
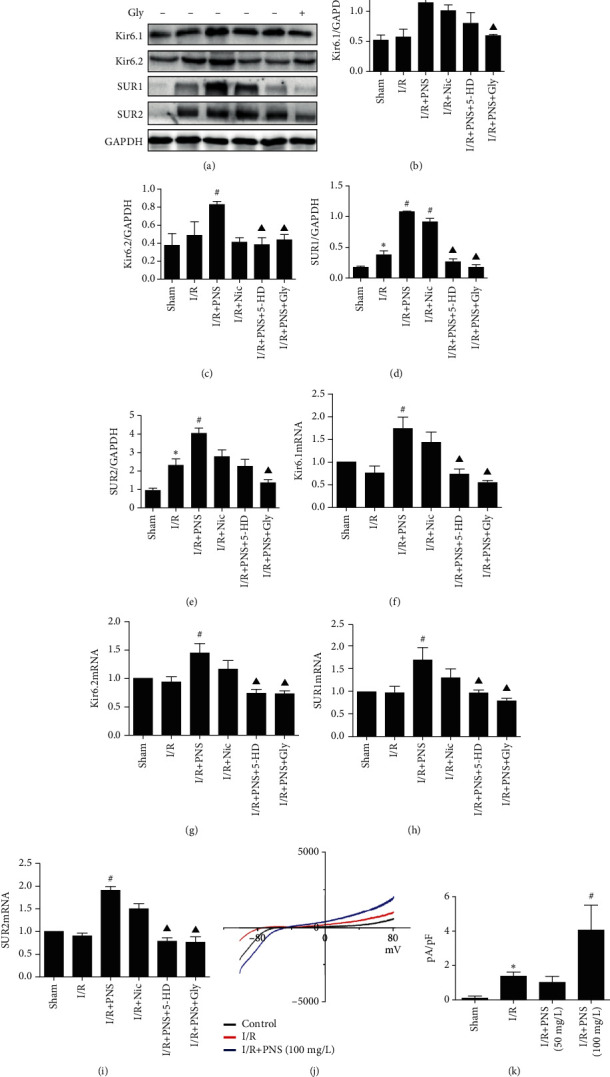
PNS pretreatment improved the expression and opening of K_ATP_ in rats with myocardial reperfusion injury. (a) Subunits of K_ATP_ in rats with myocardial reperfusion injury were measured using western blotting. (b–e) Quantitative analysis results for Kir6.1, Kir6.2, SUR1, and SUR2, respectively. (f–i) Quantitative analysis results for mRNA of Kir6.1, Kir6.2, SUR1, and SUR2 from real-time PCR, respectively. (j) Representative traces of whole-cell currents at voltage-clamp pulses at voltages ranging from −80 to 80 mV. (k) Pretreatment with PNS (50, 100 mg/L) on left ventricular cardiomyocytes at 0 mV after I/R (*n* = 5–7). ^∗^*p* < 0.05 vs. Sham, #*p* < 0.05 vs. I/R, ^▲^*p* < 0.05 vs. I/R+PNS.

We then explored the mechanism of PNS against MIRI. Considering that K_ATP_ is an important protective target of heart disease and part of an effective measure during pretreatment against MIRI, we focused on the effect of PNS in K_ATP_ on the MIRI of rats. The protein and gene expression of four subunits (Kir6.1, Kir6.2, SUR1, and SUR2) of K_ATP_ were first detected (Figure 4(a)). Western blotting indicated that the expression of four subunits was increased in the I/R group relative to the sham operation group; SUR1 and SUR2 increased significantly (*p* < 0.05, Figures 4(b)–4(e)). PNS could significantly upregulate the protein and gene expression of four K_ATP_ subunits (*p* < 0.05, Figures 4(b)–4(i)), and the effect was mostly consistent with that of Nic. However, the effect of PNS on K_ATP_ was blocked after pretreatment with 5-HD or Gly (*p* < 0.05, Figures 4(b)–4(i). Thereafter, we used the patch-clamp technique to observe the effect of PNS on the opening state of the K_ATP_ channel in the ventricular myocytes of I/R rats. The results indicated that K_ATP_ channels were opened in the I/R group, in contrast to those in the control group (*p* < 0.05), and PNS (100 mg/L) could further upregulate the potassium current mediated by the K_ATP_ channel (*p* < 0.05 vs. I/R group) Figures 4(j), 4(k). Therefore, increasing the expression and opening of K_ATP_ is one of the potential mechanisms of PNS against MIRI.

### 3.3. Cardiac Protection of PNS on MIRI-Induced Inflammation Could Be Suppressed by Gly

**Figure 5:**
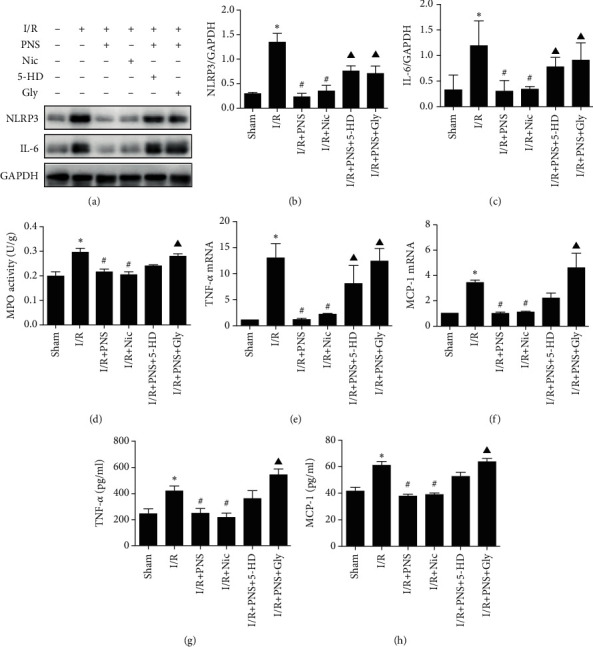
PNS inhibited the inflammatory response of reperfused myocardium. (a–c) Protein expression levels of NLRP3 and IL-6. (d) MPO activity level in the myocardium. (e–h) mRNA and protein expression levels of TNF-*α* and MCP-1. (*n* = 5–7). ^∗^*p* < 0.05 vs. Sham, #*p* < 0.05 vs. I/R, ^▲^*p* < 0.05 vs. I/R+PNS.

The inflammatory process in the development of MIRI occurs mainly through the activation of inflammatory factors. The increased production of proinflammatory cytokines, including tumour necrosis factor-*α* (TNF-*α*) and interleukin (IL)-1*α*, is a major feature of MIRI-induced inflammation [20]. TNF-*α* promotes the formation of reactive oxygen species, which in turn activates the transcription factor nuclear factor (NF-*κ*B) and induces the translocation of NF-*κ*B from the cytosol to the nucleus. We found that PNS could significantly inhibit inflammatory body NLRP3 and the classic inflammatory factor Interleukin-6 (IL-6), and the anti-inflammatory effect was inhibited by K_ATP_ blockers 5-HD and Gly (Figures 5(a)–5(c)). This anti-inflammation effect was further confirmed by the activity of myeloperoxidase (MPO), which is an independent inflammatory risk factor, represented by the evaluation index of neutrophil infiltration into tissue (Figure 5(d)). In addition, we observed that mRNA expression as well as protein level of inflammation-related TNF-*α* and MCP-1 was significantly increased by the onset of I/R, and PNS treatment inhibited these increases. By contrast, treatment with 5-HD, the selective mitoK_ATP_ inhibitor, negated the effect of PNS on resistance to MCP-1 protein expression. Similarly, glibenclamide (Gly), a nonselective inhibitor of K_ATP_, significantly reversed the protective effect of PNS (Figures 5(e)–5(h)). These results indicated that PNS plays an anti-inflammatory role in MIRI, which may be driven by K_ATP_ opening, meaning that it can be deteriorated by the inhibitors 5-HD and (especially) Gly.

### 3.4. PNS Reduced NF-*κ*B Activity, Which Was Mediated by K_ATP_

**Figure 6:**
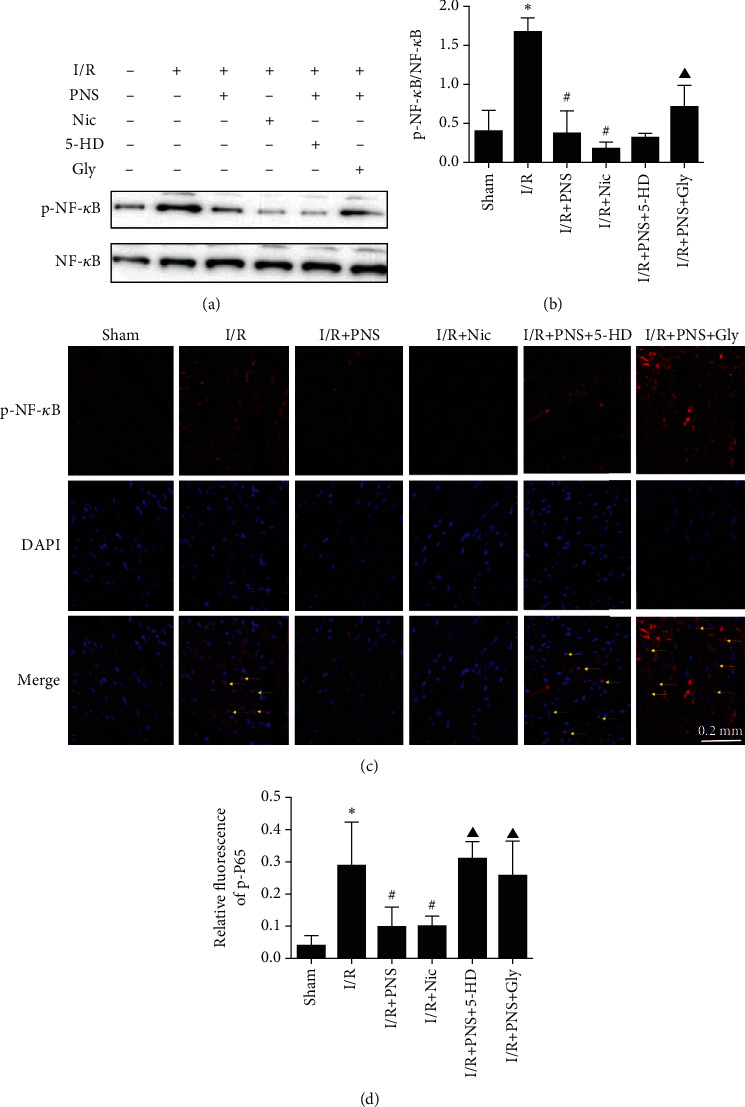
PNS inhibited NF-*κ*B phosphorylation and nuclear translocation in reperfused myocardia. (a) Protein levels of p-NF-*κ*B in cytoplasm; total NF-*κ*B level was used as a control. (b) Quantitative analysis of the ratios of p-P65/P65. (c) Immunofluorescence results indicating changes in the expression and nuclear translocation of p65 under different treatments (p65 was labelled with red fluorescence, original magnification ×400). The yellow arrow indicates that p-NF-*κ*B was colocalized with the nucleus. (d) Quantitative analysis of the fluorescence intensity of p65 in each group (*n* = 5). ^∗^*p* < 0.05, #*p* < 0.05 vs. I/R, ^▲^*p* < 0.05 vs. I/R+PNS. The primers encoding rat TNF-*α*, MCP-1, Kir6.1, Kir6.2, SUR1, SUR2, and GAPDH are presented in the following table.

To determine whether the anti-inflammation effect of PNS was mediated by K_ATP_ in MIRI, we assessed the NF-*κ*B (P65) in rat heart (Figure 6(a)). We measured the phosphorylation level of p-P65 and the level of total protein. Compared with the sham group, the phosphorylation level of p-P65 in the I/R group was significantly increased, but PNS and Nic reduced its ratio to total protein (Figure 6(b)). The ratio of p-P65 in the 5-HD group did not change, whereas the protective effect of PNS was reversed after Gly pretreatment. In addition, to further clarify whether PNS was the key medium in the inhibition of NF-*κ*B signaling, we used immunofluorescence to detect p-P65 nuclear translocation signals. The nuclear translocation of p-P65 in I/R rat myocardium was observed under a fluorescence microscope. The intensity of p-P65 in the I/R group was significantly higher than that in the sham group. However, after PNS and Nic treatment, the nuclear translocation of p-P65 decreased (Figure 6(c)). After 5-HD and Gly were used, the p-P65 nuclear signal still increased (Figure 6(d)) (*p* < 0.05).

## 4. Discussion

We studied the protective effect of PNS against inflammation in rats with MIRI. Our results showed that PNS could inhibit inflammatory response by activating K_ATP_, thus preventing MIRI.

In critical patients, LVDP and LVSP are the main determinants of cardiac function and serve as important factors for hemodynamic evaluation [21]. The maximum value of dp/dt is generally regarded as a sign of changes in the left ventricle and is traditionally used as a reliable indicator of myocardial performance [22]. Left ventricular systolic dysfunction is increasingly considered as the key phenomenon of perioperative cardiac complications in major cardiac surgery patients. In our results, we noted significant changes in the two indexes after I/R. Such changes indicated that the left ventricular function was significantly damaged after I/R, and the use of PNS reduced this damage. We found that the arrangement of myocardial fibres of H&E staining was disordered and broken, inflammatory cell infiltration was serious, resulting in cell swelling, and myocardial injury was obvious. PNS pretreatment, however, was obviously able to reverse this phenomenon. PNS could reduce the infarct size also.

Both clinical and animal experiments have demonstrated PNS had effectively cardioprotection function [23–25]. In this study, we also found that PNS could reverse MIRI-induced inflammatory response. In this process, K_ATP_ opening and expression play a key role. ATP depletion during ischemia, anoxia, and malnutrition will lead to disorder in energy metabolism. Researchers found that the K_ATP_ channel plays an important role in the drug pretreatment of MIRI [4]. K_ATP_ channel opening could increase myocardial oxygen supply and improve cardiac function and myocardial energy metabolism [26]. We found that the myocardial K_ATP_ channel mRNA level and expression of protein in MIRI rats were significantly higher than those in sham-operated rats. These findings suggest that K_ATP_ channels are activated under stress. However, the opening of K_ATP_ channels in MIRI rats was also increased when PNS pretreatment was administered, which is consistent with results on the role of Nic, a K_ATP_ channel opener. Nevertheless, the nonselective K_ATP_ channel blocker, Gly, inhibited the protective effect of PNS. This suggests that PNS may provide energy for pathological myocardium by increasing the expression and opening of K_ATP_ and then promote the improvement of cardiac function. In addition, the whole-cell patch clamp exhibited a significant increase in ATP-dependent potassium current (Ik-_ATP_) when rats were perfused with 100 mg/L PNS by the Langendorff technique. This result corroborates our assertion.

During myocardial ischemia reperfusion, increased inflammatory response can be observed in a variety of inflammatory factors [27]. NLRP3 inflammasome has been reported to play a new role in I/R rat while IL-6 is an early mediator of proinflammatory response in MIRI [28, 29]. In addition, MPO is a highly abundant protein in neutrophils [30]. In our results, PNS could obviously reduce NLRP3 and IL-6, as well as inhibited MPO. We also observed that PNS attenuated the mRNA and protein levels of TNF-*α* and MCP-1 in rat hearts, which are the most important cytokine involved in the activation of NF-*κ*B during I/R injury.

In our present results, the expression of p-NF-*κ*B was significantly increased in the I/R group, and the western blotting results were consistent with the immunofluorescence results. The expression of p-NF-*κ*B protein in the cardiomyocytes was significantly decreased upon treatment with PNS, which indicated that PNS can inhibit the activation and entry into the nucleus process of NF-*κ*B in rat myocardia following I/R. After the activation and entry of p-NF-*κ*B, the expression of downstream inflammatory factors TNF-*α* and MCP-1 proteins and genes in the I/R group significantly increased, and the protein and gene level in the PNS group were significantly lower than those in the I/R group. These findings suggest that PNS can inhibit inflammation after MIRI in rats.

Interestingly, we discovered that blocking the K_ATP_ pathway reversed the anti-inflammatory effect of PNS, which was corroborated by results for 5-HD (selective inhibitor of mitoK_ATP_) and Gly (nonselective inhibitor of K_ATP_), the pharmacological blockers of K_ATP_. After treatment with these two blockers, the protective effect of PNS was blocked, especially by Gly, which indicates that the protective effect of PNS is partly due to the opening of the K_ATP_ channel. MitoK_ATP_ is located in the inner membrane of mitochondria, whereas sarcK_ATP_ is located in the inner membrane of cells. Although some studies have reported mitoK_ATP_ to play the predominant role in this process [31], in our experiment, sarcK_ATP_ may play this role. Of course, this means that Gly could inhibit the K_ATP_ channel on both the mitochondrial and cell membrane, thus inhibiting cardioprotection from PNS.

These results indicate that the protective effect of PNS is partly due to the opening of the K_ATP_ channel. The activation of the K_ATP_ channel can prevent cell swelling and preserve cell functional integrity, thus reducing the production and release of inflammatory factors during ischemia and reperfusion. Moreover, it is likely that opening these channels is a prerequisite for PNS protective I/R damage.

## 5. Conclusion

PNS pretreatment before myocardial ischemia can reduce MPO release and improve cardiac function as well as reduce myocardial infarct size during reperfusion. The cardioprotective effect of PNS is related to the decreasing of NLRP3 inflammasome and inflammatory mediators IL-6, MCP-1, and TNF-*α* in damaged myocardia. In addition, blocking K_ATP_ channels can reverse these effects of PNS; therefore, the activation of the K_ATP_ channels is related to the cardioprotection of PNS. Since inflammatory response is crucial in the expansion of I/R injury, PNS can be introduced into beneficial drugs to reduce the incidence of myocardial reperfusion injury.

## Figures and Tables

**Table 1:** Real-time PCR primer sequence.

TNF-*α*	F	GCCAATGGCATGGATCTCAAAG	kir6.2	F	TCCAACAGCCCGCTCTAC
	R	GCCAATGGCATGGATCTCAAAG		R	GATGGGGACAAAACGCTG
MCP-1	F	AATGGGTCCAGAAGTACATTAGAAA	SUR1	F	GATGGGGACAAAACGCTG
	R	GGTGCTGAAGTCCTTAGGGTTG		R	AGCCAGCAGAATGATGACAG
kir6.1	F	GAGTGAACTGTCGCACCAGA	SUR2	F	ACCTGCTCCAGCACAAGAAT
	R	GAGTGAACTGTCGCACCAGA		R	TCTCTTCATCACAATGACCAGG
GAPDH	F	GCTGGGGCTCACCTGAAGG			
	R	TCTCTTCATCACAATGACCAGG			

## Data Availability

The data used to support the findings of this study are available from the corresponding author upon request.
